# A Calibration Method for a Laser Triangulation Scanner Mounted on a Robot Arm for Surface Mapping

**DOI:** 10.3390/s19081783

**Published:** 2019-04-14

**Authors:** Gerardo Antonio Idrobo-Pizo, José Maurício S. T. Motta, Renato Coral Sampaio

**Affiliations:** 1 Faculty of Gama-FGA, Department Electronics Engineering, University of Brasilia, Brasilia-DF 72.444-240, Brazil; gerardo.idrobo@gmail.com; 2 Faculty of Technology-FT, Department of Mechanical and Mechatronics Engineering, University of Brasilia, Brasilia-DF 70910-900, Brazil; 3 Faculty of Gama-FGA, Department Software Engineering, University of Brasilia, Brasilia-DF 72.444-240, Brazil; renatocoral@gmail.com

**Keywords:** 3D scanner calibration, laser scanning, vision triangulation, robot calibration, robotic vision, surface mapping, robotic welding, turbine blade repairing

## Abstract

This paper presents and discusses a method to calibrate a specially built laser triangulation sensor to scan and map the surface of hydraulic turbine blades and to assign 3D coordinates to a dedicated robot to repair, by welding in layers, the damage on blades eroded by cavitation pitting and/or cracks produced by cyclic loading. Due to the large nonlinearities present in a camera and laser diodes, large range distances become difficult to measure with high precision. Aiming to improve the precision and accuracy of the range measurement sensor based on laser triangulation, a calibration model is proposed that involves the parameters of the camera, lens, laser positions, and sensor position on the robot arm related to the robot base to find the best accuracy in the distance range of the application. The developed sensor is composed of a CMOS camera and two laser diodes that project light lines onto the blade surface and needs image processing to find the 3D coordinates. The distances vary from 250 to 650 mm and the accuracy obtained within the distance range is below 1 mm. The calibration process needs a previous camera calibration and special calibration boards to calculate the correct distance between the laser diodes and the camera. The sensor position fixed on the robot arm is found by moving the robot to selected positions. The experimental procedures show the success of the calibration scheme.

## 1. Introduction

In the last decade, the spread of 3D scanning devices has been increasing progressively in industry, mainly for the inspection and quality control of processes that use robotic and machine vision systems, which need motion control within an unknown workspace [1,2]. The main noncontact measurement methods include visual detection [3,4] and laser scanning methods [5].

Up to now, few works have been published about sensor model calibration describing the combination of motion control with the high positioning accuracy of industrial robots (1 mm maximum error tolerance) and 3D noncontact measuring systems [1,2,6,7,8,9,10,11,12,13,14]. A robotic system can perform measurements from different angles and directions avoiding occlusion, shading problems, and insufficient data from the surface to be measured [15,16].

To achieve an accurate measurement of an object’s pose expressed in a world coordinate system using a vision system mounted on the robot, various components need to be calibrated beforehand [11,17,18]. This includes the robot tool position, expressed in the robot base coordinate system; the camera pose, expressed in the robot tool coordinate system; and the object pose, expressed in the camera coordinate system. In recent years, there have been major research efforts to individually resolve each of the tasks above [19]. For instance, the calibration of camera and laser projectors to find the intrinsic and extrinsic parameters of the digitizer or 2D image coordinates from 3D object coordinates that are expressed in world coordinate systems [20]. In addition, there is also research focused on robot calibration in order to increase the accuracy of the robot end-effector positioning by using measures expressed in a 3D digitizer coordinate system [21]. Once all system components are individually calibrated, the object position expressed in the robot base reference system can be directly obtained from vision sensor data.

The calibration of the complete robot-vison system can be achieved from the calibration of its components or subsystems separately, taking into account that each procedure for the component calibration is relatively simple. If any one of the components of the system has its relative position modified, the calibration procedure must be repeated only for that component of the system.

Noncontact measurement systems have been analyzed and compared regarding their measurement methodology and accuracy in a comparative and analytical form in [22], considering their high sensitivity to various external factors inherent in the measuring process or the optical characteristics of the object. However, in the case of noncontact optical scanning systems and due to the complexity of the assessment to the process errors, there is no standardized method to evaluate the measurement uncertainty, as described in ISO/TS/14253-2:1999 and IEC Guide 98-3:2008, which makes it difficult to establish criteria to evaluate the performance of the measurement equipment. In ISO 10360-7:2011, for example, there is currently no specification of performance requirements for the calibration of laser scanners, fringe projection, or structured light systems.

An experimental procedure has been conceived to calibrate the relative position between the vision sensor coordinate system and the robot base coordinate system consisting of moving the robot manipulator to different poses for the digitization of a standard sphere of known radius [10]. Through a graphical visualization algorithm, a trajectory could be chosen by the user for the robot tool to follow. The calibration procedure proposed in that work agreed with the standards specifications of ISO 10360-2 for coordinate measuring machines (CMMs). A similar work is presented in [23].

In this article, a calibration routine is presented to acquire surface 3D maps from a scanner specially built with a vision camera and two laser projectors to transform these coordinates into object coordinates expressed in the robot controller for surface welding. The calibration of the geometric parameters of the vision sensor can be performed by using a flat standard block to acquire several images of the laser light at different angular positions of the mobile laser projector. The image of the fixed sensor is stored to compute the intersection between it and the images of the light projections of the mobile laser projector. The transformation of 3D maps from the sensor coordinates to the robot base coordinates was performed using a method to calibrate the sensor position fixed on the robot arm together with the geometric parameters of the robot. Results have shown that in the application the scanning sensor based on triangulation can generate 3D maps expressed in the robot base coordinates with acceptable accuracy for welding, with the values of positioning errors smaller than 1 mm in the working depth range.

## 2. The Optical System

**Figure 1:**
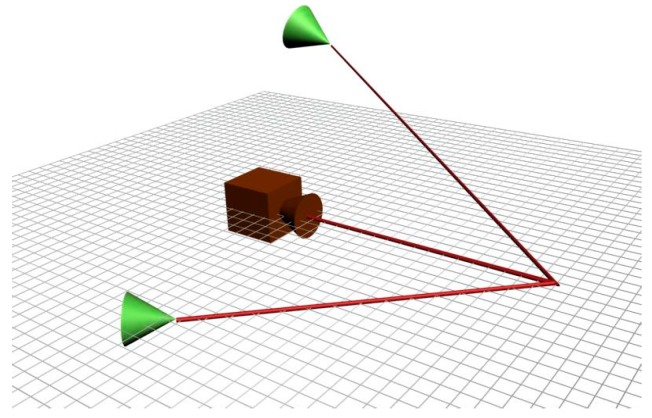
Sketch of the laser projectors and camera (VISSCAN-3D).

The surface scanning system developed in this research does not depend on positioning sensors to measure the angular displacement of the laser light source. However, the determination of this angular displacement is required for the triangulation process to produce the depth map. The proposed sensor replaces the angular displacement sensor by another laser source, such that the system is composed of two laser projectors and a camera, as shown in the sketch in Figure 1.

In addition to the use of a second laser projector, it is also necessary to include two geometrical restrictions to the mounting system. The first restriction is that the relative position between the two planes of light projected by the lasers must be perpendicular so that the triangulation equations proposed are valid. The second restriction is that one of the laser projectors is fixed. These restrictions will be discussed in detail later.

**Figure 2:**
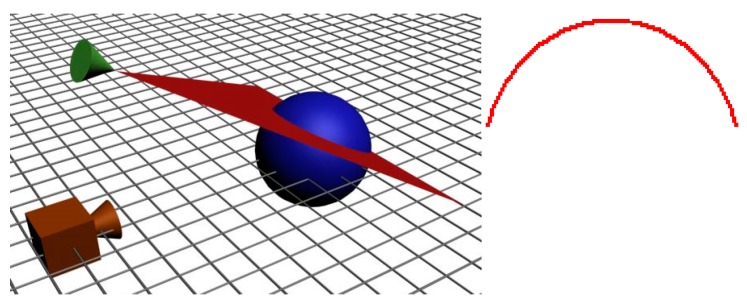
Projection of a laser light plane on a surface.

Each of the laser diodes project a light plane on a surface generating a light curve on it, as shown in Figure 2.

**Figure 3:**
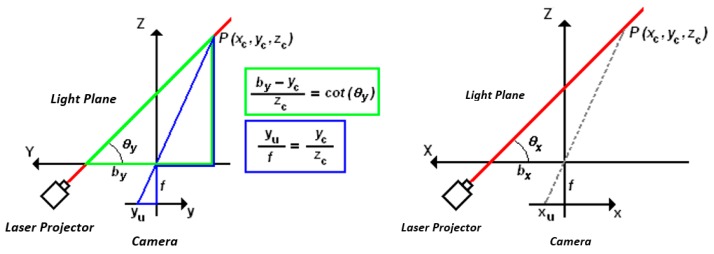
A light plane and the image formation of a point on the first and second laser line, showing the triangulation from the laser projection on the object surface.

It is considered that the light plane in Figure 2 is parallel to the X-axis of the camera coordinate system. In the Z–Y plane in Figure 3, the image of a single point, *P*, on the laser line is projected on the camera sensor such that the image formation of *P* is a projection by the central perspective model.

There are two triangles in green and blue in Figure 3 from which a relationship between the 3D coordinates of point P and the 2D image coordinates can be formulated.

So,

(1) $$\{\begin{array}{l} y_{c}=\frac{y_{u}b_{y}}{fcot\left(\theta_{y}\right)+y_{u}}\\ z_{c}=\frac{fb_{y}}{fcot\left(\theta_{y}\right)+y_{u}} \end{array},$$

where

- (*x_c_*, *y_c_*, *z_c_*) → 3D coordinates of point *P* in camera coordinates (mm);
- (*x_u_*, *y_u_*) → image coordinates of point *P* (mm);
- *b_y_* → distance along the Y-axis between the camera origin and the laser plane parallel to the X-axis;
- *θ_y_* → angle between the Y-axis and the laser plane parallel to the X-axis;
- *f* → camera focal length.

From the perspective equations, *x_c_/x_u_ = y_c_/y_u_*, it is possible to determine the value of *x_c_* from

(2) $$x_{c}=\frac{x_{u}b_{y}}{fcot\left(\theta_{y}\right)+y_{u}},$$

such that the 3D coordinates of point *P* are completely defined by the 2D image coordinates by

(3) $$\left[\begin{array}{c} x_{c}\\ y_{c}\\ z_{c} \end{array}\right]=\frac{b_{y}}{fcot\left(\theta_{y}\right)+y_{u}}\left[\begin{array}{c} x_{u}\\ y_{u}\\ f \end{array}\right]$$

Due to the restrictions of the mounting system, both laser planes are perpendicular to each other so that the second laser is parallel to the Y-axis of the camera. The equations of the first laser line can be derived from a projection of the X–Z plane shown in Figure 3 such that the image formation of point *P* on the line can be formulated with the perspective model with Equation (4):

(4) $$\{\begin{array}{l} x_{c}=\frac{x_{u}b_{x}}{fcot\left(\theta_{x}\right)+x_{u}}\\ z_{c}=\frac{fb_{x}}{fcot\left(\theta_{x}\right)+x_{u}} \end{array},$$

where

- *b_x_* → distance along the X-axis between the camera origin and the laser plane parallel to the Y-axis;
- *θ_x_* → angle between the X-axis and the laser plane parallel to the Y-axis;

From the perspective equations:

(5) $$y_{c}=\frac{y_{u}b_{x}}{fcot\left(\theta_{x}\right)+x_{u}},$$

such that the 3D coordinates of point *P* are completely defined by its 2D image coordinates using Equation (6):

(6) $$\left[\begin{array}{c} x_{c}\\ y_{c}\\ z_{c} \end{array}\right]=\frac{b_{x}}{fcot\left(\theta_{x}\right)+x_{u}}\left[\begin{array}{c} x_{u}\\ y_{u}\\ f \end{array}\right]$$

**Figure 4:**
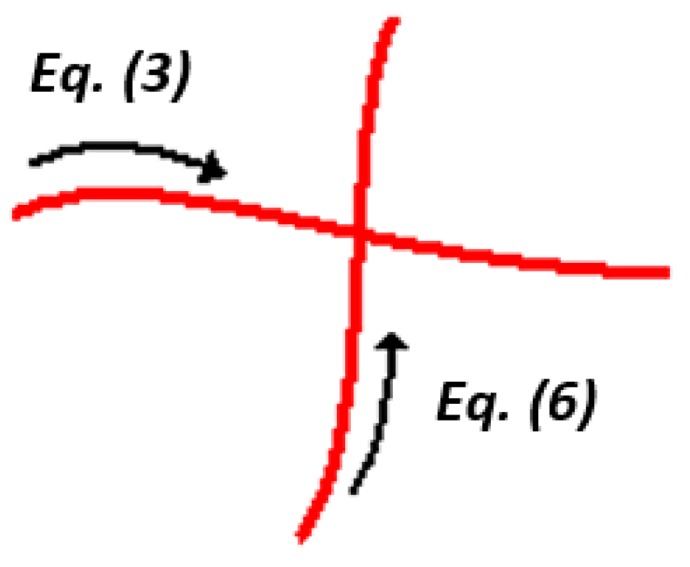
Mathematical model of the two light projections.

Equations (3) and (6) define a relationship between the 3D coordinates of a point *P* and its 2D image coordinates, but these equations are not valid for all points of the light projection. Equation (3) is valid only for one of the laser’s lines, and Equation (6) is valid only for the other, as shown in Figure 4.

However, at the point of intersection *P_int_* between the two lasers’ lines projected on the surface, both equations are valid.

From the image coordinates of the intersection point (*x_int_* and *y_int_*), the 3D coordinates of *P_int_* can be calculated from both Equations (3) and (6), so a relationship between the angular displacement of both laser diodes, *θ_x_* and *θ_y_*, can be obtained as

(7) $$\{\begin{array}{l} cot\left(\theta_{y}\right)=\frac{1}{f}\left[\frac{b_{y}}{b_{x}}\left(fcot\left(\theta_{x}\right)+x_{int}\right)-y_{int}\right]\\ cot\left(\theta_{x}\right)=\frac{1}{f}\left[\frac{b_{x}}{b_{y}}\left(fcot\left(\theta_{y}\right)+y_{int}\right)-x_{int}\right] \end{array}$$

Since one of the laser diodes has no degree of freedom, then either *cot(θ_x_)* or *cot(θ_y_)* is constant and previously known, as well as the values of *b_x_*, *b_y_*, and *f*, which are also calibrated previously. Therefore, the other term *cot*(*θ_x_*) or *cot*(*θ_y_*) of the mobile laser can be obtained from Equation (7) and Equation (3), or alternatively Equation (6) can convert the 2D image coordinates into 3D coordinates of each of the points on the line projected onto the surface by the mobile laser diode.

**Figure 5:**
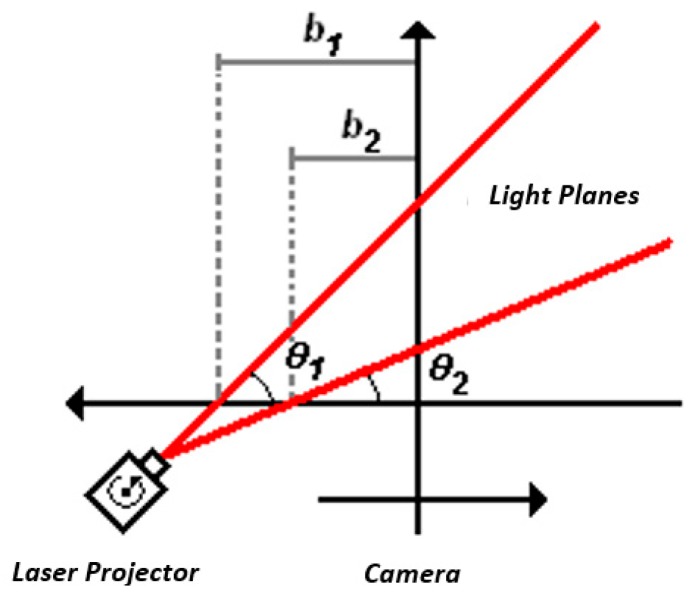
Effect of the misalignment of the mobile laser projector.

However, when rotating the mobile laser projector, the model described by Equation (3), Equation (6), and Equation (7) cannot describe the system geometry. As can be seen in Figure 5, if the mobile laser diode is not aligned with the camera’s coordinate system, the distance, *b*, does not remain constant while scanning the surface.

**Figure 6:**
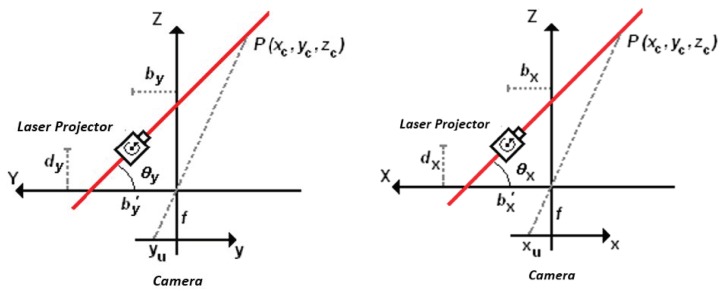
Misalignment parameters.

To consider this effect in the digitization equations, it is necessary to include a misalignment parameter, and then it is possible to perform a correction on the base distance of the laser for each angular position according to Equation (8) and Figure 6:

(8) $$\{\begin{array}{l} {b_{y}}^{\prime }=b_{y}+d_{y}cot\left(\theta_{y}\right)\\ {b_{x}}^{\prime }=b_{x}+d_{x}cot\left(\theta_{x}\right) \end{array}$$

It is important to note that although this misalignment can occur in both diodes, it generates variation only on the base distance of the mobile laser beam. For the fixed laser, regardless of the misalignment, the base distance, *b*’, remains constant. In other words, after determining this distance, no compensation is necessary due to the variation in the position of the mobile beam.

Rewriting the scanning equations, including the effect of the mobile laser misalignment, yields

(9) $$\left[\begin{array}{c} x_{c}\\ y_{c}\\ z_{c} \end{array}\right]=\frac{{b_{y}}^{\prime }}{fcot\left(\theta_{y}\right)+y_{u}}\left[\begin{array}{c} x_{u}\\ y_{u}\\ f \end{array}\right]$$

(10) $$\left[\begin{array}{c} x_{c}\\ y_{c}\\ z_{c} \end{array}\right]=\frac{{b_{x}}^{\prime }}{fcot\left(\theta_{x}\right)+x_{u}}\left[\begin{array}{c} x_{u}\\ y_{u}\\ f \end{array}\right]$$

where *b_x_*’ and *b_y_*’ are given by Equation (8) and

(11) $$\{\begin{array}{l} cot\left(\theta_{y}\right)=\frac{1}{f}\left[\frac{b_{y}}{b_{x}}\left(fcot\left(\theta_{x}\right)+x_{int}\right)-y_{int}\right]{\left[1-\frac{d_{y}}{fb_{x}}\left(fcot\left(\theta_{x}\right)+x_{int}\right)\right]}^{-1}\\ cot\left(\theta_{x}\right)=\frac{1}{f}\left[\frac{b_{x}}{b_{y}}\left(fcot\left(\theta_{y}\right)+y_{int}\right)-x_{int}\right]{\left[1-\frac{d_{x}}{fb_{y}}\left(fcot\left(\theta_{y}\right)+y_{int}\right)\right]}^{-1} \end{array}$$

**Figure 7:**
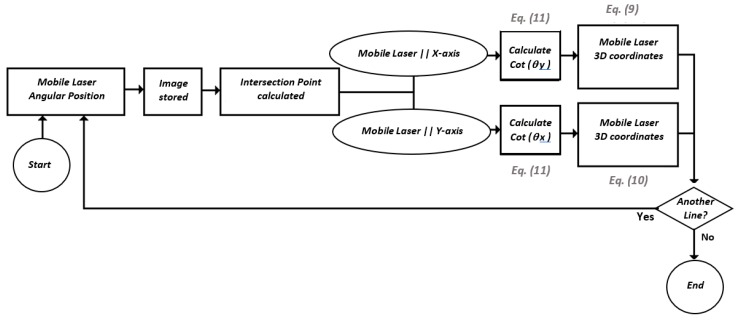
Surface scanning algorithm.

A flowchart shows each of the steps for the complete scan of a surface in Figure 7. It is important to note that the camera model and the parameters *b_x_*, *b_y_*, *d_x_*, *d_y_*, *cot*(*θ_x_*), and *cot*(*θ_y_*) are previously calibrated. Depending on which diode laser is used as the mobile laser, either Equation (9) or Equation (10) is used.

## 3. Optical System Calibration

Since the camera is calibrated, all camera intrinsic and extrinsic parameters are completely determined, and the optical system can be calibrated with these parameters.

**Figure 8:**
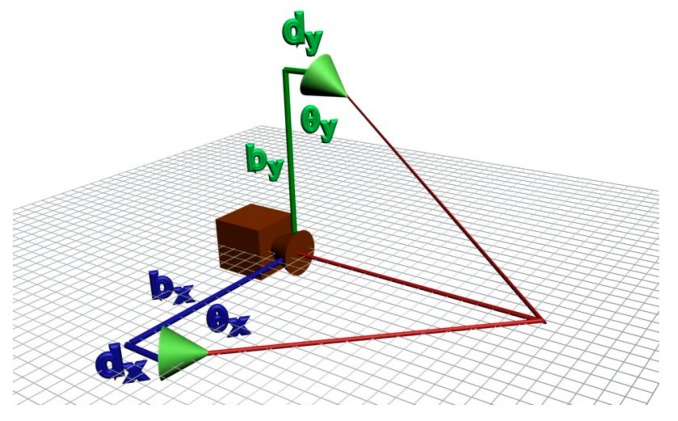
Geometric parameters of the digitization system.

The calibration of the optical system is the process of identifying the real values of the geometric parameters of the optical system described previously. These parameters can be seen in Figure 8.

A point P on the object reference system with coordinates (*x_w_, y_w_, z_w_*) has its coordinates expressed in the camera coordinate system (*x_c_, y_c_, z_c_*) by the following equation:

(12) $$\left[\begin{array}{c} x_{c}\\ y_{c}\\ z_{c} \end{array}\right]=R\cdot \left[\begin{array}{c} x_{w}\\ y_{w}\\ z_{w} \end{array}\right]+T,$$

where *R* is an orthonormal rotation matrix 3 × 3 and *T* is a translation vector representing the spatial coordinates of the origin of the world reference system expressed in the camera coordinate system.

Considering that *R* and *T*, defined in Equation (12), perform the transformation of the world reference system to the camera reference system, it is possible to determine the equation of the reference plane in relation to the camera from the transformation below:

(13) $$0x_{w}+0y_{w}+1z_{w}+0=0R,\to TAx_{c}+By_{c}+Cz_{c}+D=0,$$

where *z_w_* = 0.

To transform the normal plane vector to the camera’s coordinate system, one can use

(14) $$\left[\begin{array}{c} A\\ B\\ C \end{array}\right]=\left[\begin{array}{ccc} r_{1} & r_{2} & r_{3}\\ r_{4} & r_{5} & r_{6}\\ r_{7} & r_{8} & r_{9} \end{array}\right]\left[\begin{array}{c} 0\\ 0\\ 1 \end{array}\right]=\left[\begin{array}{c} r_{3}\\ r_{6}\\ r_{9} \end{array}\right]$$

To determine D in Equation (13) and considering that a point *[T_x_ T_y_ T_z_]^T^* belongs to the calibration plane, then

(15) $$AT_{x}+BT_{y}+CT_{z}+D=0\to D=-r_{3}T_{x}-r_{6}T_{y}-r_{9}T_{z}$$

So, the calibration plane in relation to the camera frame is completely defined as

(16) $$Ax_{c}+By_{c}+Cz_{c}+D=0,$$

where $A=r_{3}$, $B=r_{6}$, $C=r_{9}$, and $D=-r_{3}T_{x}-r_{6}T_{y}-r_{9}T_{z}$.

**Figure 9:**
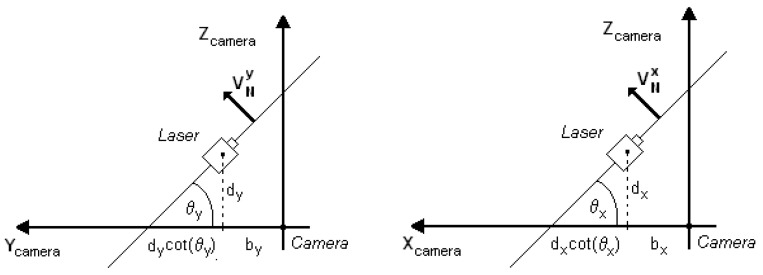
Generated light planes and the geometric parameters.

The next step is the determination of the planes generated by each of the laser beams, as shown in Figure 9, where $V_{N}^{X}$ and $V_{N}^{Y}$ represent the normal vectors of the generated planes, and $P_{L}^{X}$ and $P_{L}^{Y}$ are the positions of the laser diodes related to the camera.

The equations of these planes are given by

(17) $$\{\begin{array}{c} V_{N}^{Y}=\left[\begin{array}{ccc} 0 & 1 & cot\left(\theta_{y}\right) \end{array}\right]\\ P_{L}^{Y}=\left[\begin{array}{ccc} 0 & b_{y} & d_{y} \end{array}\right] \end{array}\Rightarrow y_{c}+cot\left(\theta_{y}\right)z_{c}-b_{y}-d_{y}cot\left(\theta_{y}\right)=0$$

(18) $$\{\begin{array}{c} V_{N}^{X}=\left[\begin{array}{ccc} 1 & 0 & cot\left(\theta_{x}\right) \end{array}\right]\\ P_{L}^{X}=\left[\begin{array}{ccc} b_{x} & 0 & d_{x} \end{array}\right] \end{array}\Rightarrow x_{c}+cot\left(\theta_{x}\right)z_{c}-b_{x}-d_{x}cot\left(\theta_{x}\right)=0$$

The intersection of these planes with the calibration board can be determined through Equations (16)–(18). These intersections are the projections of the laser light on the board surface and are mathematically described as lines in space.

The plane defined by Equation (17) is

(19) $$\{\begin{array}{c} Ax_{c}+By_{c}+Cz_{c}+D=0\\ y_{c}+cot\left(\theta_{y}\right)z_{c}-b_{y}-d_{y}cot\left(\theta_{y}\right)=0 \end{array}$$

By choosing *x_c_* as a free parameter, the solution of the system is given from the parametric equation of the line of intersection between these planes:

(20) $$\{\begin{array}{c} x_{c}=t\\ y_{c}=\frac{Acot\left(\theta_{y}\right)}{C-Bcot\left(\theta_{y}\right)}x_{c}+\frac{Cb_{y}+Cd_{y}cot\left(\theta_{y}\right)+Dcot\left(\theta_{y}\right)}{C-Bctg\left(\theta_{y}\right)}\\ z_{c}=\frac{-A}{C-Bcot\left(\theta_{y}\right)}x_{c}+\frac{-Bb_{y}-Bd_{y}cot\left(\theta_{y}\right)-D}{C-Bcot\left(\theta_{y}\right)} \end{array}$$

Similarly, for the plane of light described by Equation (18):

(21) $$\{\begin{array}{c} Ax_{c}+By_{c}+Cz_{c}+D=0\\ x_{c}+cot\left(\theta_{x}\right)z_{c}-b_{x}-d_{x}cot\left(\theta_{x}\right)=0 \end{array}$$

(22) $$\{\begin{array}{c} x_{c}=\frac{Bcot\left(\theta_{x}\right)}{C-Acot\left(\theta_{x}\right)}y_{c}+\frac{Cb+Cd_{x}cot\left(\theta_{x}\right)+Dcot\left(\theta_{x}\right)}{C-Acot\left(\theta_{x}\right)}\\ y_{c}=t\\ z_{c}=\frac{-B}{C-Acot\left(\theta_{x}\right)}y_{c}+\frac{-Ab-Ad_{x}cot\left(\theta_{x}\right)-D}{C-Acot\left(\theta_{x}\right)} \end{array}$$

The existence of the free parameter, *t*, in the equations of the intersection between the planes is to avoid divisions by zero since it is possible that the values of *x_c_* and *y_c_* are constant in light planes parallel to the X and Y axes, respectively.

Thus, the image coordinates (*x_im_* and *y_im_*) from a point on the laser line, since this point is on the plane of the calibration board, are obtained, then the coordinates of this point (*x_c_*, *y_c_*, and *z_c_*) relative to the camera reference system can be obtained using the camera model equations proposed by Tsai [24], Lenz, and Tsai [25], referred to as Radial Alignment Constraint (RAC model), with some modifications proposed by Zhuang and Roth [26], comprising the equations below together with the equation of the calibration board:

(23) $$\{\begin{array}{l} \frac{x_{im-}C_{x}}{1-kr^{2}}=f_{x}\frac{x_{c}}{z_{c}}\\ \frac{y_{im-}C_{y}}{1-kr^{2}}=f_{y}\frac{y_{c}}{z_{c}}\\ Ax_{c}+By_{c}+Cz_{c}+D=0\\ r^{2}=\mu^{2}{\left(x_{im}-C_{x}\right)}^{2}+{\left(y_{im}-C_{y}\right)}^{2} \end{array},$$

where (*C_x_, C_y_*) are the coordinates of the image center in pixels, *μ* = *fy/fx*, *k* = coefficient of image radial distortion, *kr*^2^ << 1, and *fx* and *fy* are the focal length in pixels corrected for the shape of the pixel dimensions in the X and Y axes, respectively (scale factors *sx* and *sy* in Table 1, where *fx* = *f*/*sx* and *fy* = *f*/*sy*).

Solving the system above, the coordinates (*x_c_*, *y_c_*, and *z_c_*) of a point of the laser line can be obtained directly as

(24) $$\{\begin{array}{l} x_{c}=\frac{-A_{x}D}{AA_{x}+BB_{y}+C}\\ y_{c}=\frac{-B_{y}D}{AA_{x}+BB_{y}+C}\\ z_{c}=\frac{-D}{AA_{x}+BB_{y}+C} \end{array},$$

where

(25) $$\{\begin{array}{c} A_{x}=\frac{1}{f_{x}}\frac{x_{ℑ}-C_{x}}{1-kr^{2}}\\ B_{y}=\frac{1}{f_{y}}\frac{y_{ℑ}-C_{y}}{1-kr^{2}}\\ r^{2}=\mu^{2}{\left(x_{ℑ}-C_{x}\right)}^{2}+{\left(y_{ℑ}-C_{y}\right)}^{2} \end{array}$$

Therefore, using these obtained coordinates (*x_c_*, *y_c_*, and *z_c_*) and the equation of the projection line of the laser plane in space (Equations (20) and (22)) it is possible to obtain a linear system of equations for b,cot(θ), and dcot(θ):

(26) $$\left[\begin{array}{ccc} Ax_{c}+By_{c}+D & C & C\\ Bz_{c} & -B & -B \end{array}\right]\left[\begin{array}{c} cot\left(\theta_{y}\right)\\ b_{y}\\ d_{y}cot\left(\theta_{y}\right) \end{array}\right]=\left[\begin{array}{c} Cy_{c}\\ Ax_{c}+Cz_{c}+D \end{array}\right],$$

(27) $$\left[\begin{array}{ccc} Ax_{c}+By_{c}+D & C & C\\ Az_{c} & -A & -A \end{array}\right]\left[\begin{array}{c} cot\left(\theta_{x}\right)\\ b_{x}\\ d_{x}cot\left(\theta_{x}\right) \end{array}\right]=\left[\begin{array}{c} Cx_{c}\\ By_{c}+Cz_{c}+D \end{array}\right]$$

It is easily seen that Columns 2 and 3 are identical in Equation (27), i.e., regardless of the number of points used the system will always have a rank of 2. Therefore, the misalignment parameters, *d_x_* and *d_y_*, cannot be obtained directly from these systems.

For the calibration of *d_x_* and *d_y_*, two or more positions of the mobile laser are used and the values of *d.cot(θ)* and *b* are determined at once. The systems of Equation (26) and Equation (27) can be modified to

(28) $$\left[\begin{array}{cc} Ax_{c}+By_{c}+D & C\\ Bz_{c} & -B \end{array}\right]\left[\begin{array}{c} cot\left(\theta_{y}\right)\\ {b_{y}}^{\prime } \end{array}\right]=\left[\begin{array}{c} Cy_{c}\\ Ax_{c}+Cz_{c}+D \end{array}\right]$$

(29) $$\left[\begin{array}{cc} Ax_{c}+By_{c}+D & C\\ Az_{c} & -A \end{array}\right]\left[\begin{array}{c} cot\left(\theta_{x}\right)\\ {b_{x}}^{\prime } \end{array}\right]=\left[\begin{array}{c} Cx_{c}\\ By_{c}+Cz_{c}+D \end{array}\right],$$

where

(30) $$\{\begin{array}{l} {b_{x}}^{\prime }=b_{x}+d_{x}cot\left(\theta_{x}\right)\\ {b_{y}}^{\prime }=b_{y}+d_{y}cot\left(\theta_{y}\right) \end{array}$$

For the solution of these systems, a single point of the laser line is sufficient; however, the use of several points on the laser line and the optimization based on least squares or the singular value decomposition (SVD) can produce more accurate results.

Therefore, with N different positions of the mobile laser, it is possible to determine the actual base distance of the laser diode and its misalignment value through an overdetermined system, calibrating the laser parameters completely:

(31) $$\{\begin{array}{c} b_{x}+d_{x}{}^{1}cot\left(\theta_{x}\right)={}^{1}{b_{x}}^{\prime }\\ b_{x}+d_{x}{}^{2}cot\left(\theta_{x}\right)={}^{2}{b_{x}}^{\prime }\\ \vdots \\ b_{x}+d_{x}{}^{N}cot\left(\theta_{x}\right)={}^{N}{b_{x}}^{\prime } \end{array}$$

(32) $$\{\begin{array}{c} b_{y}+d_{y}{}^{1}cot\left(\theta_{y}\right)={}^{1}{b_{y}}^{\prime }\\ b_{y}+d_{y}{}^{2}cot\left(\theta_{y}\right)={}^{2}{b_{y}}^{\prime }\\ \vdots \\ b_{y}+d_{y}{}^{N}cot\left(\theta_{y}\right)={}^{N}{b_{y}}^{\prime } \end{array}$$

For the calibration of the fixed laser, the same procedure is performed; however, since the angle of inclination of the fixed laser is constant, the determination of the apparent base distance, *b*’, is sufficient.

**Figure 10:**
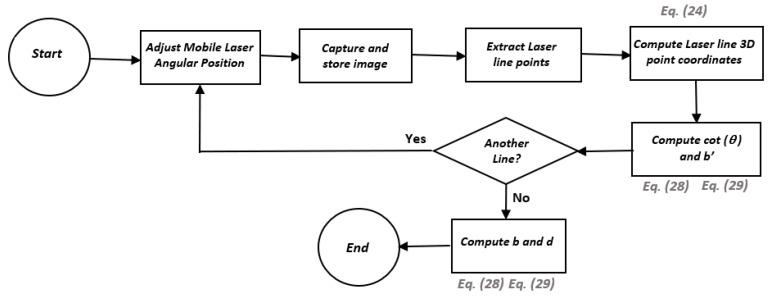
Mobile laser calibration algorithm.

**Figure 11:**
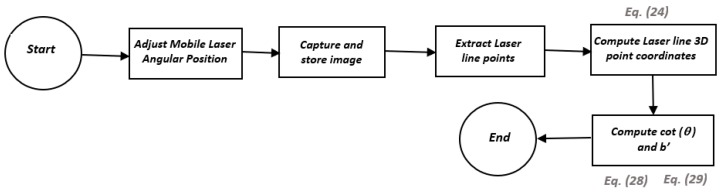
Fixed laser calibration algorithm.

The entire calibration of the optical system can be summarized through the algorithms illustrated in Figure 10 and Figure 11.

## 4. Calibration of the Sensor Position

The 3D laser scanning sensor based on triangulation developed in this research is intended to produce 3D maps of the surfaces of hydraulic turbine blades. The sensor must be mounted and fixed on the robot arm to be moved over the surfaces by the robot. Therefore, for the 3D coordinates of the map to be assigned with respect to the controller coordinate system at the robot base, the sensor needs to have its position and orientation expressed in the robot base coordinate system previously determined.

The process to determine the sensor position is accomplished by moving the robotic arm with the sensor attached on it over a gage block of known dimensions, such that the range images represented in the respective 3D camera coordinates are obtained and recorded. Subsequently, the robot has its end-effector (weld torch) positioned at various point positions on the gage block surface and the robot coordinates are recorded and related to the coordinates of the same position point expressed in the camera coordinate system of the map.

From several point positions, the transformation between the camera coordinate system and the robot base coordinate system can be obtained and used in the parameter identification routine described in the next sections.

### 4.1. Robot Forward Kinematic Model

**Figure 12:**
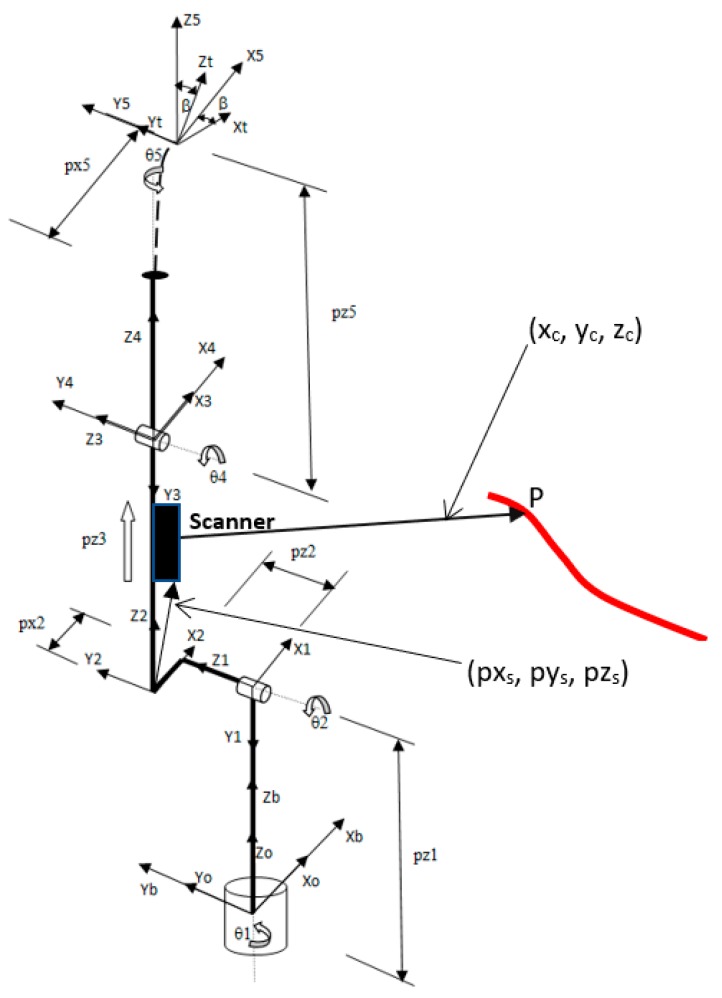
Robot at zero position with joint coordinate systems, link variables, point P, and sensor position vectors.

Considering the robot model shown in Figure 12, homogeneous transformation matrices that relate coordinate frames from the robot base (b) to the robot torch/tool (t) can be formulated as follows:

(33) Ttb=T0b∗T10∗T21∗T32∗T43∗T54∗Tt5=[nxoxaxpxnynz0oyoz0ayaz0pypz1],

where Ti+1i is the homogeneous transformation between two successive joint coordinate frames.

The transformations shown in Equation (33) can be formulated with only 4 elementary motions as proposed by the Denavit–Hartenberg (D–H) convention [27] as below:

(34) Tii−1=Rz(θ)∗Tz(d)∗Tx(l)∗Rx(α)=Tii−1=[cos(θ)−(cos(α)∗sin(θ))sin(α)∗sin(θ)sin(θ)cos(α)∗cos(θ)−(cos(α)∗sin(θ))00sin(α)0cos(α)0l∗cos(θ)l∗sin(θ)d1],

where *θ* and *α* are rotation parameters in Z and X axes, respectively, and *d* and *l* are translation parameters along the Z and X axes, respectively. The application of Equation (34) to each of the consecutive robot joint frames by using the geometric parameters shown in Figure 12 produces the general homogeneous transformation of the manipulator.

The entries of the general manipulator transformation, T50, according to Equation (33), excluding the rotation of the torch tip coordinate frame by the angle *β* (Figure 12), are formulated below, as the robot forward kinematic equations:

(35) nx=−sin(θ1)·sin(θ5)+cos(θ5)·cos(θ1)·cos(θ2+θ4)

(36) ox=−sin(θ1)·cos(θ5)−sin(θ5)·cos(θ1)·cos(θ2+θ4)

(37) ax=cos(θ1)·sin(θ2+θ4)

(38) $$\begin{array}{c} px=pz3\cdot cos\left(\theta 1\right)\cdot sin\left(\theta 2\right)+px2\cdot sin\left(\theta 1\right)\cdot sin\left(\theta 2\right)-pz2\cdot sin\left(\theta 1\right)\\ +pz5\cdot cos\left(\theta 1\right)\cdot sin\left(\theta 2+\theta 4\right)\\ +px5\cdot \left[cos\left(\theta 1\right)\cdot cos\left(\theta 2+\theta 4\right)\right]-sin\left(\theta 1\right)\cdot sin\left(\theta 5\right) \end{array}$$

(39) ny=cos(θ1)·sin(θ5)+cos(θ5)·sin(θ1)·cos(θ2+θ4)

(40) oy=cos(θ1)·cos(θ5)−sin(θ5)·sin(θ1)·cos(θ2+θ4)

(41) ay=sin(θ1)·sin(θ2+θ4)

(42) $$\begin{array}{c} py=pz3\cdot sin\left(\theta 1\right)\cdot sin\left(\theta 2\right)-px2\cdot sin\left(\theta 1\right)\cdot cos\left(\theta 1\right)+pz2\cdot cos\left(\theta 1\right)\\ +pz5\cdot sin\left(\theta 1\right)\cdot sin\left(\theta 2+\theta 4\right)\\ +px5\cdot [cos\left(\theta 1\right)\cdot sin\left(\theta 5\right)\\ +cos\left(\theta 5\right)\cdot sin\left(\theta 1\right)\cdot cos\left(\theta 2+\theta 4\right)] \end{array}$$

(43) nz=−sin(θ2+θ4)·cos(θ5)

(44) oz=sin(θ2+θ4)·sin(θ5)

(45) az=cos(θ2+θ4)

(46) $$\begin{array}{c} pz=pz1+pz5\cdot cos\left(\theta 2+\theta 4\right)+pz3\cdot cos\left(\theta 2\right)+px2\cdot sin\left(\theta 2\right)\\ -px5\cdot sin\left(\theta 2+\theta 4\right)\cdot cos\left(\theta 5\right) \end{array}$$

### 4.2. Parameter Identification Modeling

Robot calibration is a process of fitting a nonlinear complex model consisting of a parametrized kinematic model with error parameters to experimental data. The error parameters are identified by minimizing an error function [17].

A robot kinematic model consists of a set of nonlinear functions relating joint variables and link geometric parameters to the robot end-effector pose, such as in

P = T_1_. T_2_…T_n_, (47)

where T_i_ are any link transformations defined in Equation (34), P is the manipulator transformation and *n* is the number of links. If the kinematic model uses a convention of 4 elementary transformations per link, like the D–H convention, the manipulator pose error can be expressed as (from Equation (34))

(48) $$\Delta P=\frac{\partial P}{\partial \theta }\Delta \theta +\frac{\partial P}{\partial \alpha }\Delta \alpha +\frac{\partial P}{\partial d}\Delta d+\frac{\partial P}{\partial l}\Delta l,$$

where *θ*, *α*, *d*, and *l* are geometric parameters that relate a robot joint frame to the next joint frame, where *d* and *l* are translation parameters, and *θ* and *α* are rotation parameters in two of the three coordinate axes, respectively.

**Figure 13:**
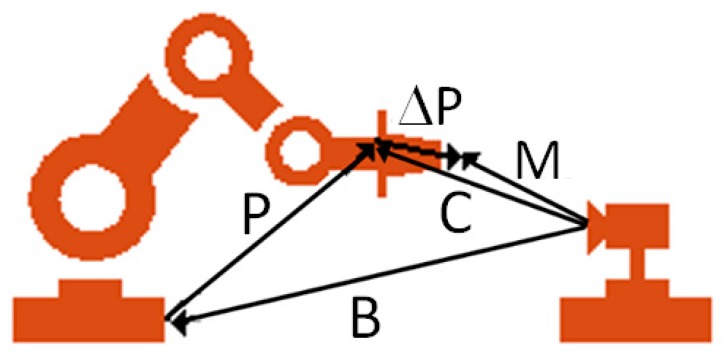
Calibration transformations [28].

The derivatives shown in Equation (48) characterize the partial contribution of each of the geometric error parameters of each joint, consisting of the total pose error of the robot’s end-effector, which can be measured with proper measuring devices. Considering the measured robot poses (M) and the transformation from the measurement system frame to the robot base (B), ΔP is the vector shown in Figure 13.

The transformation, B, can also be considered as a virtual link belonging to the robot model that must be identified. So, the pose error, ΔP, can be calculated with Equation (49) as [28]

(49) ΔP=M−P−B=M−C

The manipulator transformation, P, is updated each time a new set of geometric error parameters is fitted through an iterative process, and, when the calibration process finishes, P is the minimum deviation of the measured poses.

Equation (48) can be rewritten in a matrix form for *m* measured poses in the form of a Jacobian matrix comprising the partial derivatives of P, such that Δ*x* is the vector of the model parameter errors as in Equation (49):

(50) [ΔP1ΔP2⋮ΔPm]=[∂P1∂θ∂P1∂α∂P1∂d∂P1∂l∂P2∂θ∂P2∂α∂P2∂d∂P2∂l⋮⋮⋮⋮∂Pm∂θ∂Pm∂α∂Pm∂d∂Pm∂l]·[ΔθΔαΔdΔl]=[J1J2⋮Jm]·Δx⇒J·Δx=ΔP

The Jacobian matrix size depends on the number of measured poses in the robot workspace (*m*) and on the number of error parameters in the model (*n*). The matrix order is η*m* x *n*, such that η is the number of space degrees of freedom (3 position and 3 orientation parameters). Then, the calibration problem can be set as the solution of the nonlinear system **J.**x = **b**.

A widely used method to solve this type of system is the Squared Sum Minimization (SSM). Several other methods are discussed extensively with their related algorithms in [22]. A successful method for the solution of nonlinear least squares problems in practice is the Levemberg–Marquardt algorithm. Many versions of this algorithm have proved to be globally convergent. The algorithm is an iterative solution method with few modifications of the Gauss–Newton method to reduce numerical divergence problems.

### 4.3. Algorithm for the Transformation of Coordinates from the Sensor to the Robot Base 

**Input:** The matrix with all the coordinates of the map points scanned by the sensor in a scan, expressed in the sensor coordinate system. Each point coordinate is transformed to coordinates represented in the robot base coordinate system with the homogeneous transformation equations below:

A0P **=** A01 * A12 * A2S * ASP, (51)

where,

A0P = matrix representing the position of the scanned object point (P) in the robot base coordinate system (0);

A01 = matrix representing the position of Joint 1 (1) in the robot base coordinate system (0);

A12 = matrix representing the position of Joint 2 (2) in the Joint 1 coordinate system (1);

A2S = matrix representing the position of the sensor (S) in the Joint 2 coordinate system (2) (px_s_, py_s_, and pz_s_) (see Figure 12);

ASP = matrix representing the position of the scanned point (P) in the sensor coordinate system (S) (x_c_, y_c_, and z_c_) (see Figure 12).

The homogeneous transformations are shown below:

(52) $$A01=\left[\begin{array}{cccc} cos\left(\theta_{1}\right) & -cos\left(\alpha_{1}\right)sin\left(\theta_{1}\right) & sin\left(\alpha_{1}\right)sin\left(\theta_{1}\right) & px_{1}cos\left(\theta_{1}\right)\\ sin\left(\theta_{1}\right) & cos\left(\alpha_{1}\right)cos\left(\theta_{1}\right) & -sin\left(\alpha_{1}\right)cos\left(\theta_{1}\right) & px_{1}sin\left(\theta_{1}\right)\\ 0 & sin\left(\alpha_{1}\right) & cos\left(\alpha_{1}\right) & pz_{1}\\ 0 & 0 & 0 & 1 \end{array}\right]$$

(53) $$A12=\left[\begin{array}{cccc} cos\left(\theta_{2}\right) & -cos\left(\alpha_{2}\right)sin\left(\theta_{2}\right) & sin\left(\alpha_{2}\right)sin\left(\theta_{1}\right) & px_{2}cos\left(\theta_{2}\right)\\ sin\left(\theta_{2}\right) & cos\left(\alpha_{2}\right)cos\left(\theta_{2}\right) & -sin\left(\alpha_{2}\right)cos\left(\theta_{1}\right) & px_{2}sin\left(\theta_{2}\right)\\ 0 & sin\left(\alpha_{2}\right) & cos\left(\alpha_{2}\right) & pz_{2}\\ 0 & 0 & 0 & 1 \end{array}\right]$$

(54) $$A2S=\left[\begin{array}{cccc} cos\left(\theta_{3}\right) & -cos\left(\alpha_{3}\right)sin\left(\theta_{3}\right) & sin\left(\alpha_{3}\right)sin\left(\theta_{3}\right) & px_{s}cos\left(\theta_{3}\right)-py_{s}sin\left(\theta_{3}\right)\\ sin\left(\theta_{3}\right) & cos\left(\alpha_{3}\right)cos\left(\theta_{3}\right) & -sin\left(\alpha_{3}\right)cos\left(\theta_{3}\right) & py_{s}cos\left(\theta_{3}\right)+px_{s}sin\left(\theta_{3}\right)\\ 0 & sin\left(\alpha_{3}\right) & cos\left(\alpha_{3}\right) & pz_{3}\\ 0 & 0 & 0 & 1 \end{array}\right]$$

(55) $$ASP=\left[\begin{array}{cc} \begin{array}{cc} 1 & 0\\ 0 & 1 \end{array} & \begin{array}{cc} 0 & x_{c}\\ 0 & y_{c} \end{array}\\ \begin{array}{cc} 0 & 0\\ 0 & 0 \end{array} & \begin{array}{cc} 1 & z_{c}\\ 0 & 1 \end{array} \end{array}\right],$$

where symbols are described in Section 4.1 and:

θ1 = Joint 1 position when scanning, recorded from the robot controller; 

θ2 = Joint 2 position when scanning, recorded from the robot controller; 

pz3 = Joint 3 position when scanning, recorded from the robot controller; 

(x_c_, y_c_, z_c_) = object point coordinates, P, represented in the sensor coordinate system. 

The constant parameters were previously determined from a robot calibration process, and details about the calibration process of this robot can be seen in [28]. The pertinent results are listed below: 

α1 = −89.827°; 

α2 = 90°; 

pz1 = 275 mm; 

pz2 = 104.718 mm; 

pz3 = joint variable position in the controller +103.677 mm;

px1 = −0.059 mm;

px2 = 33.389 mm;

θ1 = joint variable position in the controller + 0.1097°; 

θ2 = joint variable position in the controller + 89.602°. 

The parameters to be identified are px_s_, py_s_, and pz_s_, and the results of the identification routine are presented in the homogeneous transformation A2S:

(56) $$A2S=\left[\begin{array}{cccc} -0.00087266 & -0.00204203 & -0.99999753 & -29.92711162\\ -0.99999962 & 0.00000178 & -0.00087266 & 94.9961525\\ 0 & 0.99999792 & -0.00204203 & pz_{3}\\ 0 & 0 & 0 & 1 \end{array}\right]$$

**Output:** The matrix with all the object point coordinates of a scan expressed in the robot base coordinate system. These coordinate values must be input into the robot controller so that, through the forward kinematics, the robot torch reaches the programmed trajectory points.

## 5. Results and Discussion

### 5.1. Sensor Calibration

**Figure 14:**
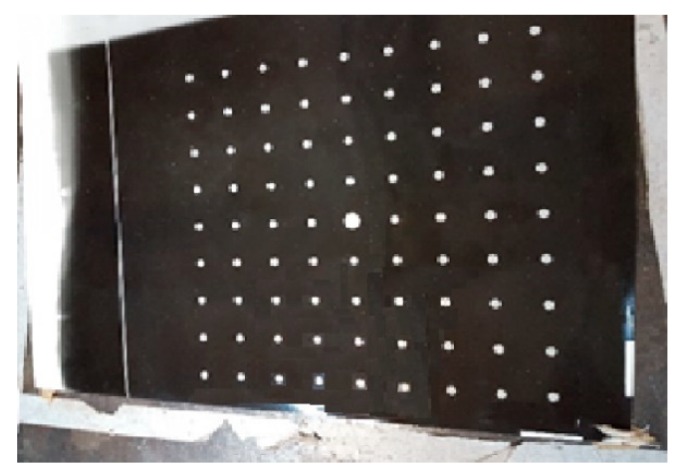
Calibration board made of photographic paper printed with light through etched glass used to calibrate the camera intrinsic parameters, with 9 × 9 dots of 26.5 ± 0.1 mm from each other.

**Figure 15:**
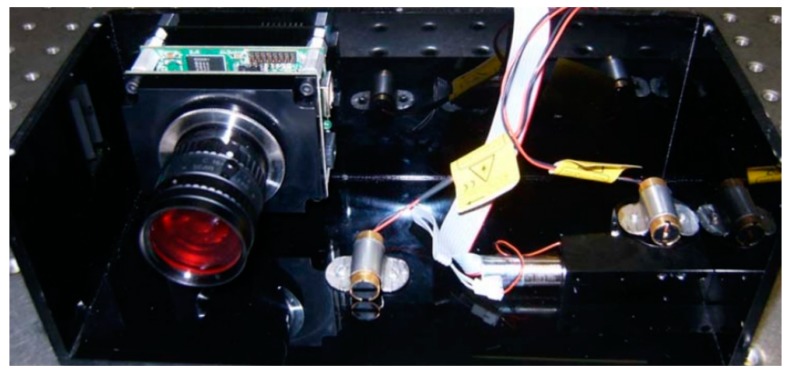
Scanner with camera and two laser light projectors.

A calibration board (see Figure 14) was used to first calibrate the camera intrinsic parameters, such as the focal length (*f*), image center (Cx and Cy), scale parameters (sx and sy), and an image radial distortion factor (*k*). After the camera was calibrated, the geometric parameters of the sensor (Figure 8) could be calibrated from several images acquired from the laser plane projection on a plane board. The sensor with the camera, lens, and laser light projectors can be seen in Figure 15.

The algorithm used to calibrate the camera is based on the RAC model proposed by Tsai and Lenz [25]. Data from the camera calibration process reveal a distance from the target to the camera of approximately 383 mm. Table 1 shows the camera intrinsic parameter results obtained with the calibration routine.

### 5.2. Calibration of the Sensor Position

**Figure 16:**
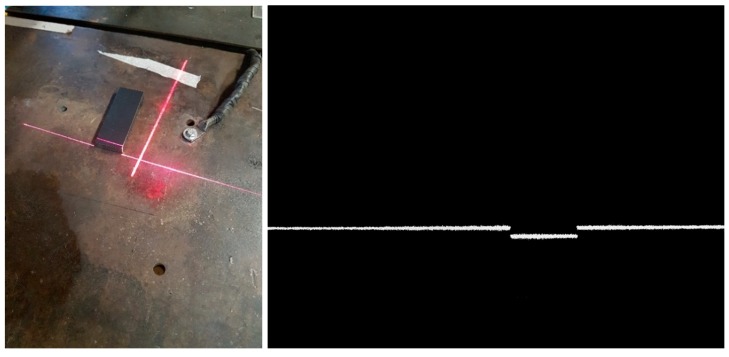
Image of the standard block and processed image data in a laser projector position.

The vision sensor was mounted on the robot arm according to Figure 12. The calibration of the sensor geometric parameters was performed using a flat plate and a gage block for depth verification (100 × 50 × 10 mm), through several images of laser light lines in various positions of the mobile laser projector. The light line image of the fixed sensor reflected on the metal plate must be vertical when projected on the screen and can be stored during each test to be subsequently used to calculate the projection of the light lines emitted by the mobile sensor on the plate. Images from the calibration process can be seen in Figure 16.

The geometric parameters that must be obtained with the parameter identification routine discussed in Section 4.2 and Section 4.3 to calibrate the sensor are shown in Figure 8. The experimental results from the sensor calibration procedures can be seen in Table 2.

### 5.3. Accuracy Evaluation of the Robot Positioning Using the Surface 3D Sensor Map

**Figure 17:**
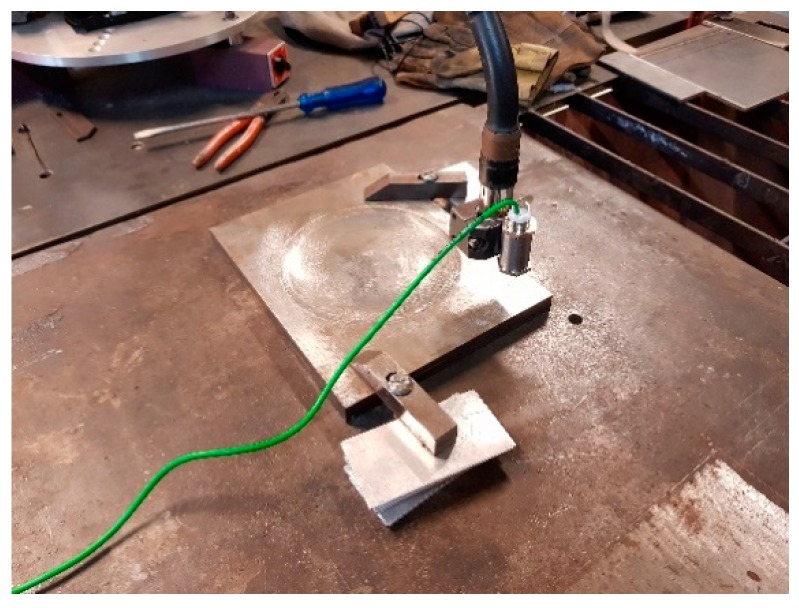
Scanning process of a metallic block.

**Figure 18:**
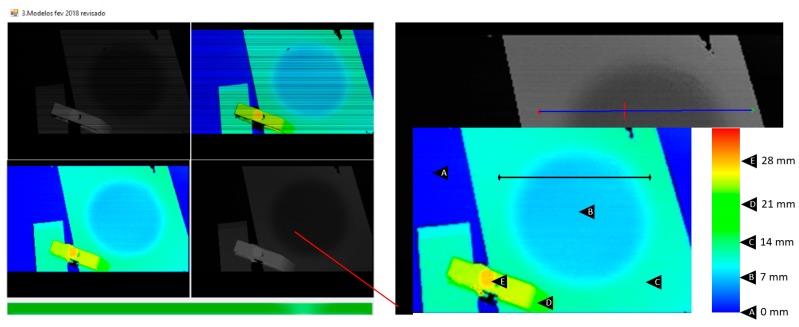
3D map of a scanned block and a programmed robot trajectory for welding.

**Figure 19:**
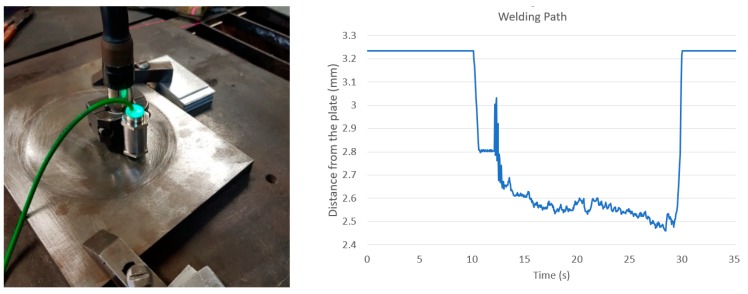
Positioning measurements along a trajectory crossing the metallic block.

**Figure 20:**
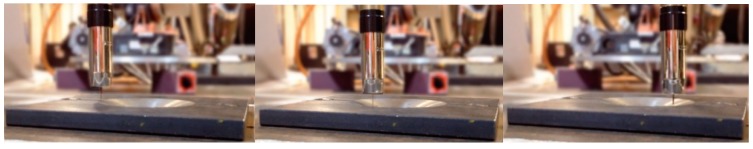
Three robot welding torch positions along a trajectory.

To evaluate the accuracy of the surface 3D maps constructed by the vision sensor and expressed on the robot base coordinate system, some tests were carried out on scanning the surfaces and positioning the robot’s end-effector (in this case an inductive proximity sensor) on the surface trajectories to be followed by the robot. In Figure 17, a metallic 3D block with known dimensions is shown. The block was scanned, and the map is shown in Figure 18. Figure 19 shows a positioning measurement with an inductive proximity sensor along a straight trajectory and the measurements are shown. Figure 20 shows the same trajectory adjusted to the welding torch.

It can be seen from the results that the proximity sensor showed instability when the distance from the surface varied and stability when the distance remained constant. This is because the sensor head has a diameter of 18 mm with a working distance that has to be within the range 0.4–4 mm, which could not be achieved when moving over the curved borders of the depressed surface of the block. However, along the flat surface, it could be observed that the sensor head could measure a distance from the surface from 2.5 to 2.6 mm, which obviously suffices welding requirements.

It was observed that, within the operating distance range of 350 to 500 mm, there was a systematic translation between the origins of the robot and the map coordinates in the X, Y, and Z axes that could easily be fixed with a simple transformation matrix, resulting in a very good accuracy in tracking the programmed trajectory.

## 6. Conclusions

This work proposed a calibration method of a laser triangulation scanner mounted on a robot arm to produce 3D surface maps expressed in the robot coordinates to be used in welding tasks on the surfaces of turbine blades. The method assumes that the robot and the camera are previously calibrated. The vision sensor embeds two laser line projectors to scan the surface in such a way that a triangulation process can construct a 3D surface map after the geometric parameters of the sensor are identified. The position of the fixed sensor on the robot arm is then calibrated and the 3D map can have its coordinates expressed in the robot base coordinate system. With the map available it is possible to perform the offline programming of robot welding tasks.

Experimental tests were performed to evaluate the accuracy of the 3D map expressed in the robot controller coordinates by moving the robot’s end-effector along a trajectory programmed over a metal block with a surface depression similar to those found in the field such that the stand-off should be kept constant. The distance from the robot end-effector and the plate surface along the trajectory was measured with a magnetic proximity sensor mounted on the robot welding torch. Results showed an average accuracy of 0.3 mm on a displacement of approximately 180 mm.

This calibration system proposal opens up an alternative to use triangulation-based laser scanners with enough accuracy in applications where the distance from the target is large but within a depth range where calibration has been performed, exactly as the application for which this system was developed.

## Figures and Tables

**Table 1:** Camera intrinsic parameters ^1^.

Focal Length f (mm)	Image Center (Cx,Cy) (pixel)	Scale Factors (sx,sy) (pixel/mm)	Radial Distortion Factor k
**9.43773**	(738, 585)	(227.27, 227.27)	9.4604 × 10^−9^

^1^ Camera CMOS Lumenera LW230—1616 × 1216, 4.4 microns squared pixels.

**Table 2:** Geometric parameters of the laser projectors in the vision sensor after calibration.

Rotatory Laser Projector		Fixed Laser Projector	
b_y_ (mm)	d_y_ (mm)	Bx’ (mm)	cot(θx)
101.973	−16.0156	−6.6788	−0.0922

## References

[1] Pérez L., Rodríguez Í., Rodríguez N., Usamentiaga R., García D. (2016). Robot Guidance Using Machine Vision Techniques in Industrial Environments: A Comparative Review. Sensors (Basel).

[2] Morozov M., Pierce S.G., MacLeod C.N., Mineo C., Summan R. (2018). Off-line scan path planning for robotic NDT. Measurement.

[3] He B.X., Li C.-L., Li J.-P., Zhang Y., Liu R.-L. (2015). Integration of intelligent measurement and detection for sealing rings used in aerospace systems. Opt. Precis. Eng..

[4] Shi Y., Sun C., Wang P., Wang Z., Duan H. (2012). High-speed measurement algorithm for the position of holes in a large plane. Opt. Lasers Eng..

[5] Kondo Y., Hasegawa K., Kawamata H., Morishita T., Naito F. (2012). On-machine non-contact dimension-measurement system with laser displacement sensor for vane-tip machining of RFQs. Nuclear Instrum. Methods Phys. Res. A Accel. Spectrom. Detect. Assoc. Equip..

[6] Heeshin K. (2016). Study on Synchronization for Laser Scanner and Industrial Robot. Int. J. Sci. Eng. Appl. Sci..

[7] Hatwig J., Reinhart G., Zaeh M.F. (2010). Automated task planning for industrial robots and laser scanners for remote laser beam welding and cutting. Prod. Eng..

[8] Craig J.J. (2005). Introduction to Robotics, Mechanics and Control.

[9] Niola V., Rossi C., Sergio S., Salvatore S. (2010). A method for the calibration of a 3-D laser scanner. Robot. Comput.-Integr. Manuf..

[10] Ren Y., Yin S., Zhu J. (2012). Calibration technology in application of robot-laser scanning system. Opt. Eng..

[11] Li J., Chen M., Jin X., Chen Y., Dai Z., Ou Z., Tang Q. (2011). Calibration of a multiple axes 3-D laser scanning system consisting of robot, portable laser scanner and turntable. Opt. Int. J. Light Electron. Opt..

[12] Tzafestas S.G., Raptis S., Pantazopoulos J. (1996). A Vision-Based Path Planning Algorithm for a Robot-Mounted Welding Gun. Image Process. Commun..

[13] Hatwig J., Minnerup P., Zaeh M.F., Reinhard G. An Automated Path Planning System for a Robot with a Laser Scanner for Remote Laser Cutting and Welding. Proceedings of the IEEE International Conference on Mechatronics and Automation.

[14] Shirinzadeh B., Teoh P.L., Tian Y., Dalvand M.M., Zhong Y., Liaw H.C. (2010). Laser interferometry-based guidance methodology for high precisión positioning of mechanisms and robots. Robot. Comput.-Integr. Manuf..

[15] Larsson S., Kjellander J.A.P. (2004). An industrial robot and a laser scanner as a flexible solution towards an automatic system for reverse engineering of unknown objects. Proceedings of the 7th Biennial Conference on Engineering Systems Design and Analysis 2004.

[16] Umeda K., Ikushima K., Arai T. 3D shape recognition by distributed sensing of range images and intensity images. Proceedings of the 1997 IEEE International Conference on Robotics and Automation.

[17] Zhuang H., Roth Z.S., Sudhakar R. (1994). Simultaneous robot/world and tool/flange calibration by solving homogeneous transformation equations of the form AX = YB. IEEE Trans. Robot. Autom..

[18] Zhuang H., Wang K., Roth Z.S. (1998). Simultaneous calibration of a robot and a hand-mounted camera. IEEE Trans. Robot. Autom..

[19] Yu C., Xi J. (2018). Simultaneous and on-line calibration of a robot-based inspecting system. Robot. Comput.-Integr. Manuf..

[20] David A.F., Jean P. (2011). Computer Vision: A Modern Approach.

[21] Larsson S., Kjellander J.A.P. (2006). Motion control and data capturing for laser scanning with an industrial robot. Robot. Auton. Syst..

[22] Barbero B.R., Ureta E.S. (2011). Comparative study of different digitization techniques and their accuracy. Comput.-Aided Des..

[23] Shen C., Zhu S. A Robotic System for Surface Measurement Via 3D Laser Scanner. Proceedings of the 2012 International Conference on Computer Application and System Modeling-ICCSM2012.

[24] Tsai R.Y. (1987). A Versatile Camera Calibration Technique for High-Accuracy 3D Machine Vision Metrology Using Off-the Shelf TV Cameras and Lenses. IEEE Int. J. Robot. Autom..

[25] Lenz R.K., Tsai R.Y. Techniques for Calibration of the Scale Factor and Image Center for High Accuracy 3D Machine Vision Metrology. Proceedings of the IEEE International Conference on Robotics and Automation.

[26] Zhuang H., Roth Z.S. (1996). Camera-Aided Robot Calibration.

[27] Denavit J., Hartenberg R.S. (1955). A kinematic notation for lower-pair mechanisms based on matrices. J. Appl. Mech..

[28] Motta J.M.S.T., Llanos-Quintero C.H., Sampaio R.C. (2016). Optimization of a Five-D.O.F. Robot for Repairing the Surface Profiles of Hydraulic Turbine Blades. Int. J. Adv. Robot. Syst..

